# Bridging Language Barriers in Healthcare: An Innovative Curriculum for Teaching Medical Students to Work Effectively with Medical Interpreters

**DOI:** 10.12688/mep.20473.1

**Published:** 2025-06-18

**Authors:** Sarah Yentz, Margaret Dobson, Jennifer Stojan, Kathleen Bronson Dussán

**Affiliations:** 1 University of Michigan Medical School, Ann Arbor, Michigan, 48109, USA

**Keywords:** Limited English Proficiency, Interpreter Use, Medical Student education

## Abstract

**Introduction:**

The proportion of limited English proficiency (LEP) patients continues to rise and now represents 10% of the US population. These patients face many health care challenges and health disparities. To address the needs of educating the next generation of physicians on how to work with patients with limited English proficiency, we developed an instructional session focused on this goal.

**Methods:**

First year medical students participated in a 90-minute session focused on best practices for working with interpreters and LEP patients. During the didactic part of the session, faculty led students in a discussion regarding best practices through use of video examples. During the interactive exercise, students obtained the HPI from a mock patient fluent in a language other than English through use of a phone interpreter.

**Results:**

Following the session, the proportion of students that felt confident in obtaining a HPI from a LEP patient increased from 27% to 72%. The proportion of students who felt that it was unacceptable to have a family member interpret for a LEP patient increased from 50.5% to 68.5%.

**Discussion:**

Our innovative and practical curriculum can be easily incorporated into medical school curricula, ensuring medical students are prepared to work with trained professional interpreters and meet the needs of a diverse patient population.

## Introduction

There has been a dramatic increase in international migration over the past five decades. There are an estimated 281 million individuals living outside their birth countries ^1^. The U.S. accounts for roughly one-fifth of the world’s migrants ^2^. According to Eurostat Statistics, 5.1 million immigrants entered the European Union (EU) from non-EU countries in 2022, a large increase in migration ^3^. This has led to language diversity within the EU, the U.S. and other immigrant destinations. For example, there are 67.3 million U.S. residents speaking a language other than English ^4^. Per the U.S. Census Bureau, anyone older than 5 years old who reports speaking English less than “very well” is considered “limited English proficiency” (LEP) ^5^. The population of limited English proficiency increased by 80% from 1990 to 2010 and now represents at least 10% of the population ^5^.

The LEP population faces many health care challenges and health disparities, including increased hospital stay lengths, unplanned readmissions, medication errors and decreased patient satisfaction. Contrarily, LEP patients’ satisfaction, comprehension, and outcomes are improved when cared for by bilingual providers and/or professional interpreters ^6, 7^. Additionally, studies suggest that the more comfortable a provider is when working with a LEP patient, the higher the physician satisfaction is with the encounter ^8^. In an environment where physician burn-out is high, medical schools should do all they can to prepare students for all types of medical encounters.

Unfortunately, the use of trained professional interpreters is low ^9^. A recent study found that professional interpretation was used for only 36% of communications with limited English proficiency patients ^10^.

Recent work on improving provider competence with medical interpretation has focused on post-graduate level learners ^11, 12^. There is less published on improving medical student competence in working with medical interpreters. Medical students believe it is a priority to learn about working with patients who are immigrants/migrants and using medical interpreters, but they feel ill-prepared to do so ^13, 14^.

Various programs have developed classes to train medical learners on the importance of interpreter use ^11, 15– 17^. To our knowledge, no other program has taught both didactics with a same-day interactive opportunity to interview a non-English-speaking mock patient through a trained professional interpreter. Mock patients are frequently used in medical education to allow learners to practice skills in communication. These interactions allow for learning at a higher level on Kirkpatrick Model, allowing students to demonstrate what they have learned. In addition, it has been shown that “just-in-time” teaching leads to more successful mastery of material ^18^. By allowing for immediate practice or observation of skills used for working with an interpreter, we are hopeful this session could create more long-lasting learning of the material.

To address the needs of educating the next generation of physicians on how to work with patients with limited English proficiency, we developed an instructional session focused on this goal. The session specifically focused on teaching skills for working with an interpreter as well as an interactive exercise to practice these skills with a trained professional interpreter.

## Methods

### Curricular context

Our educational session was part of the broader longitudinal “Doctoring” curriculum at the University of Michigan Medical School. Doctoring is a four-year course taught in small groups of 10–12 students and facilitated by two physician faculty members. This course focuses on teaching both clinical skills as well as addressing content including ethics, health disparities, bias, social justice, and humanism in medicine.

The “Working with an Interpreter” session was a 90-minute session during one of the “Doctoring” small group sessions in the first year of medical school. The 90-minute session was divided into 2 parts: 60 minutes of didactics/discussion regarding best practices for working with an interpreter and a 30-minute interactive exercise with a mock patient and phone interpreter. Prior to the session, students were required to read the student packet and read the pre-work article on appropriate use of medical interpreters ^19^. Faculty were required to read the faculty packet and pre-work article from the student packet.

### Actor and interpreter recruitment

We recruited mock patients who were fluent in another language. We had 1 fluent speaker for each group of 10–12 students. The mock patients had no formal instruction. We advised them to pretend that they do not speak English and to wait for the phone interpreter to interpret the students’ questions before answering. We provided them with the chief complaint for each case and advised that they could improvise any other history as the goal of the session was not necessarily to arrive at a diagnosis, but rather to obtain the history through an interpreter.

The University of Michigan Interpreter Services department provided interpretation over a speaker-phone free of charge. Languages used were Spanish, Russian, Korean, and Arabic.

### Session logistics

*Working with and Interpreter Didactics:* During the didactic part of the session, faculty led students in a discussion regarding best practices for working with an interpreter through use of video examples.

A video focused on how to work with an interpreter. The initial part of the video portrayed several practices that are not recommended when working with an interpreter including:

- Physician speaking to the interpreter rather than the patient.
- Interpreter using 3rd person language to describe patient complaints ("he has stomach pain") rather than first person language ("I have stomach pain").
- Interpreter not interpreting some questions from the physician and just answering on his/her own without patient input.

After watching this portion of the video, the students/faculty discussed what areas could be improved.

The students then watched the next part of the video which portrayed a “good” example of a physician working correctly with an interpreter. This video corrected the above deficiencies as well as included:

- Introduction from the interpreter prior to beginning the session.
- Example of the interpreter adding additional information about a commonly used Russian medication that is not available in the US and a natural supplement, also not commonly used in the US.

The final part of the video was information from the interpreter regarding best practices. This video included a description of consecutive vs simultaneous interpretation, a reminder that some words have no direct translation, as well as recommendations such as:

- Speak to the patient directly and use short and complete sentences.
- Ask just one question at a time.
- Let the interpreter know in advance if bad news is going to be relayed during the encounter.
- Be respectful of natural therapies that may be more prevalent in other cultures.
- Be mindful that some concepts like refills may not be common in other cultures/countries.

After the conclusion of the video, students and faculty discussed these recommendations. Faculty were invited to share examples of working with interpreters from their own clinical experiences. Faculty were encouraged to discuss why it is preferred to use professional interpreters rather than family members for interpretation. This discussion included the risks of using a family member to interpret, including:

- Concerns about patient confidentiality
- Lack of familiarity with medical terminology
- Potential to introduce bias into the conversation.
- Potential to avoid interpreting questions about intimate or sensitive topics.

*Interactive Exercise: Working with a phone interpreter:* After reviewing the didactics on best practices for working with an interpreter, one to two students were given the opportunity to practice these skills. Each student was assigned a chief complaint. Options for chief complaints were knee pain, cough, and abdominal pain.

Mock patients fluent in a language other than English (Spanish, Korean, Russian, Arabic) presented with the above chief complaint. Mock patients did not receive any formal training but were provided with several clinical factors to support the history. They were given permission to adjust and elaborate the case, as the goal of the exercise was to work with an interpreter and was not to focus on history taking skills. The cases are listed below.

(1) Right knee pain after running

a. No prior episodes, no pain/swelling in other joints

(2) Cough

a. 1 week duration, no fever, no rhinorrhea, non-smoker, no sore throat

(3) Abdominal Pain

a. Pain started 2 days ago. It is in middle of the abdomen but has been more right sided as of late. Patient has not had a bowel movement in past 2 days. No nausea/vomiting. Doesn’t feel like eating. Has not had pain like this before. Tried acetaminophen without relief.

Prior to the activity, we coordinated with the hospital interpreter services to have interpreters available for the educational activity. Faculty called the hospital interpreter services line to obtain a phone interpreter in the language of the mock patient. Faculty put the call on speaker phone to allow observing students to hear the entire interaction. Each student conducted the interview (history of present illness (HPI) only) in English via use of the phone interpreter.

Each student was given 10–15 minutes to interview the patient while the rest of the class observed. The group had a 5–10 minute debrief at the conclusion of the 30-minute session to review what went well and what was easier/harder than expected. The observing students provided feedback to the student who conducted the interview.

### Learner assessment and consent

This study “HUM00229293” was conducted in accordance with ethical standards and was reviewed and approved by the University of Michigan Institutional Review Board (IRB) on January 12, 2023. The IRB granted this study exempt status due to the nature of the research, which involved the use of anonymous survey data collected from voluntary participants. As a result, the requirement for written informed consent was waived.

Participants were informed that their involvement in this study was entirely voluntary, and choosing to participate, or not, would have no impact on their grades or standing within the institution. The survey was introduced to participants clearly stating the voluntary nature of participation and ensuring the confidentiality and anonymity of the data collected. Consent to participate in the study was implied by the completion of the survey, with participants choosing to engage with either a paper evaluation form or an online survey link, as per their preference.

All data obtained from the evaluations were kept anonymous, ensuring participant confidentiality was strictly maintained throughout the study. The evaluation results were analyzed collectively, with no individual identifying information recorded or reported. The research team is committed to maintaining the highest ethical standards in conducting research and respects all rights and dignity of the participants involved.

## Results

107/170 first year medical students (63%) filled out the pre/post evaluation in 2022 and 154/170 completed the evaluation in 2023 (90%).

We obtained data about our students’ prior experience with medical interpretation and their exposure to languages other than English. Thirty-nine percent of our students were fluent in a language other than English. Fifty-three percent grew up in a household where a language other than English was spoken. Twenty-one percent of students had interpreted for a family member during a medical appointment.

In our cohort, 93% of respondents felt it was a moderate or high priority to have content about working with an interpreter in the curriculum.

As shown in Table 1, following the session, more students understood that, if possible, family members should not serve as interpreters for LEP patients (p<0.05). Prior to the session, 50.5% of students felt it was unacceptable or slightly unacceptable to have a family member interpret for a patient who does not speak English. After participating in the session, this proportion increased to 68.5%.

**Table 1.** Pre and Post curriculum Survey Results (N=261).

Skill	Pre-curriculum (mean)	Post-curriculum (mean)	p
How acceptable is it to have a family member interpret for a LEP patient ^a^	2.69	2.18	<.00001
Confidence in obtaining the HPI from a LEP patient using an interpreter ^b^	2.78	3.8	<.00001

^a^1=unacceptable 2=slightly unacceptable, 3=neutral, 4=slightly acceptable, 5=acceptable ^b^1=not at all confident, 2=slightly confident, 3=somewhat confident, 4=moderately confident, 5=extremely confident

Students’ confidence in obtaining the HPI from an LEP patient using an interpreter also increased significantly (p<0.05). Prior to the session 27% of students felt extremely or moderately confident in obtaining a HPI for a patient who requires an interpreter. After participating in the session, this proportion increased to 72%.

The post curriculum survey contained open-ended questions whereby learners could provide feedback on the session. Representative comments included: “Incredibly helpful and informative for our future encounters.” "Looking forward to incorporating these ideas into my future practice." “Very important topics. Loved the practical advice.”

## Discussion

Accrediting bodies like the Accreditation Council for Graduate Medical Education (ACGME) have established requirements regarding cultural competency training for medical learners ^20^. Learning to successfully work with interpreters to overcome language barriers can improve health disparities. To this goal, we designed and implemented a session about best practices for working with interpreters and limited-English proficiency patients for first year medical students.

The session was well received by both the students and faculty. The didactics and video provided a framework for how to conduct a patient visit through a trained professional interpreter, i.e., speak directly to the patient, ask one question at a time, use short sentences. The curriculum also focused on the importance of not engaging family members as interpreters. After the session there was a significant increase in the proportion of students who understood that, if possible, family members should not serve as interpreters for LEP patients.

The strength of this curriculum was the interactive “just-in-time” practice of the skills discussed via a phone interpreter and a mock patient. Having the immediate opportunity to practice the skills taught in the session’s curriculum was well received. While some students participated in obtaining the ‘History of Present Illness’ (HPI), the rest of the small group could observe the flow, challenges, and successes of working with a professional interpreter and offer feedback to the learner. The success of this experience led to 72% of the students feeling extremely or moderately confident in obtaining a HPI for a patient who requires an interpreter, versus 27% prior to the session.

Our curriculum has several limitations. We utilized a self-assessment survey as the primary outcome measure. Our survey response rate for the first year the curriculum was run was only 63%. As a result, our data may not be representative of the entire 1 ^st^ year class. In addition, we are not able to measure the impact of the curriculum on students’ future clinical skills or behaviors. Finally, only one to two out of the 12 students in each small group were able to participate in interviewing the non-English speaking patient through a phone interpreter. With the resources within the University Hospital System, we were able to do this without cost of the interpreter services, and we recognize that other programs may not have that collaboration.

In the future we plan to have in person, rather than phone interpreters for the session. During the two years we ran the program, use of in-person interpretation was prohibited due to COVID restrictions. While phone interpretation worked well, we anticipate that in person interpretation may have additional benefits such as: no need to wait on hold for an available interpreter and the ability of the interpreter to pick up on patient attitudes/mannerisms. In addition, we would like to have more students actively participate with the trained professional interpreters.

Our innovative and practical curriculum meets the goals of teaching medical students the skills to work successfully with trained professional interpreters, while giving them an immediate opportunity to implement this with non-English speaking patients. It can be replicated for other medical students and residents to establish effective ways to communicate through a professional interpreter. Continued opportunities, like our curriculum, will help with decreasing language barriers, increase students’ cultural competency, and hopefully lead to decreased health disparities in their future medical practice.

## Ethics statement

This study “HUM00229293” was conducted in accordance with ethical principles outlined in the Declaration of Helsinki or comparable ethical standards. The research protocol was reviewed and approved on January 12, 2023 by the Institutional Review Boards (IRB) of the University of Michigan Medical School.

## Consent statement

Because of the nature of our study, the IRB granted exemption status for our project and written informed consent was waived given the study’s use of anonymous data. Documentation of consent was made by way of their participation in the survey. Participants were told that the survey was voluntary and would not affect their grade or standing with the school. Participating students filled out a pre and post session evaluation either by paper evaluation form or an online survey link.

## Data Availability

Harvard Dataverse: “Bridging Language Barriers in Healthcare: An Innovative Curriculum for Teaching Medical Students to Work Effectively with Medical Interpreters.” **https://doi.org/10.7910/DVN/RXPT2U ^21^** The project contains the following underlying data: MS Excel Spreadsheet - “Doctoring-Student Survey-Healthcare for Patients who are Immigrants_Migrants. (Responses)” PDF – “Appendix A-Student Packet PDF – “Appendix B-Faculty Packet PDF – “Appendix D-Student Survey Data are available under the terms of the Creative Commons Zero "No rights reserved" data waiver (CC0 1.0 Public domain dedication).
