# Supplementary material for: Bridging Language Barriers in Healthcare: An Innovative Curriculum for Teaching Medical Students to Work Effectively with Medical Interpreters
Source: MedEdPublish (2016). 2025 Jun 18;15:26. [Version 1] doi: 10.12688/mep.20473.1 (PMC12579306; doi:10.12688/mep.20473.1)
Supplement: Peer review history

# Reviewer report: Reviewer response for version 1

**DOI:** 10.21956/mep.21911.r43745

**Published:** 2025-10-31

**Authors:** Megan M. Mroczkowski

**Affiliations:** 1 Columbia University Irving Medical Center, New York, USA

SUMMARY OF MANUSCRIPT:

 The proportion of limited English proficiency patients is increasing and now comprises 10% of the United States population. In the University of Michigan Doctoring curriculum, a four-year course of 10-12 medical students facilitated by two physician faculty members, a 90-minute session titled "Working with an Interpreter" was studied using medical student survey self-report. The course contained teaching on how to work with an interpreter, video examples on both recommended and non-recommended practices, and best practices for interpreter use. 107/170 (63%) of medical students completed the pre-and-post evaluation in 2022 and 154/170 students completed the pre-and-post evaluation in 2023. 93% of respondents felt it was a moderate or high priority to have content about working with an interpreter in the curriculum. Confidence in obtaining an history of present illness with an interpreter increased from 27% to 72% after participation in the session.

 OVERALL FEEDBACK:

 This manuscript is beautifully written and clearly describes the rationale for including this important session in the medical school curriculum. All medical professionals would benefit from teaching in best practices in working with interpreters. Often, teaching in this area is ad hoc and therefore not standardized. Including this in the medical school curriculum is an elegant means to teach this important topic in a standardized manner.

Is the rationale for developing the new method (or application) clearly explained?

Yes

Is the description of the method technically sound?

Yes

Are the conclusions about the method and its performance adequately supported by the findings presented in the article?

Yes

If any results are presented, are all the source data underlying the results available to ensure full reproducibility?

Yes

Are sufficient details provided to allow replication of the method development and its use by others?

Yes

Reviewer Expertise:

Graduate medical education; Pediatric Psychiatric emergencies; child forensic psychiatry

I confirm that I have read this submission and believe that I have an appropriate level of expertise to confirm that it is of an acceptable scientific standard.

# Reviewer report: Reviewer response for version 1

**DOI:** 10.21956/mep.21911.r43743

**Published:** 2025-10-17

**Authors:** Jordan White

**Affiliations:** 1 Brown University, Providence, Rhode Island, USA

This article is a well-written summary of an instructional session designed for first year medical students to learn best practices in working with interpreters to care for patients with limited English proficiency (LEP). The session, integrated into the first year curriculum, utilizes a discussion guide, a video showing "good" and "bad" examples of medical interpretation, and the opportunity to practice (or observe a fellow student) taking a history while using a telephone interpreter.

 Student surveys showed that after this session, significantly more students felt confident in obtaining a history from a patient with LEP; significantly more students also felt that it was not acceptable to have a family member serve as an interpreter. These results were striking especially because only one student per small group practiced with the mock patient/interpreter, and the rest observed. If time allowed, having more students practice would probably be especially powerful, but it appears that observing alone is quite helpful!

 Given the increasing need for physicians skilled in working with interpreters as well as the need to address healthcare access challenges in patients with LEP, this curriculum offers a well-designed and well-received method by which to engage medical students in this important area early in their careers.  It appears from the discussion that this session was also well-received by small group leaders - it would be helpful to see results or comments from these faculty as well as the students. Overall, a great educational opportunity and a great summary paper here.

 Only a few additional questions/comments:

 1 - The faculty and student guides are available and are very helpful to others who may wish to replicate the session. I wonder if the video may also be available externally?

 2 - I was glad that the survey asked demographic questions about the students' own backgrounds (e.g., fluent in another language, having themselves interpreted for family members). It would be interesting to separate responses to understand how these relevant personal experiences may impact the use of interpreters and/or working with patients with LEP.

 3 - I appreciate the comments on next steps to potentially incorporate practice with in-person interpreters.

 4 - Just a note that the student and faculty guides use the term "cultural humility" as a way to expand "cultural competence", and also describe the use of person-first language.  It would be nice if the manuscript was consistent and used the same recommended approach and definitions (I noticed the term cultural competence as well as the term "LEP patients" rather than "patients with LEP" or "patients who speak a different language from the language spoken by the provider.")

 Great work!

Is the rationale for developing the new method (or application) clearly explained?

Yes

Is the description of the method technically sound?

Yes

Are the conclusions about the method and its performance adequately supported by the findings presented in the article?

Yes

If any results are presented, are all the source data underlying the results available to ensure full reproducibility?

Yes

Are sufficient details provided to allow replication of the method development and its use by others?

Yes

Reviewer Expertise:

Public health and medical education

I confirm that I have read this submission and believe that I have an appropriate level of expertise to confirm that it is of an acceptable scientific standard.
